# Association Between Clinical Characteristics and Short-Term Outcomes in Adult Male COVID-19 Patients With Mild Clinical Symptoms: A Single-Center Observational Study

**DOI:** 10.3389/fmed.2020.571396

**Published:** 2021-01-05

**Authors:** Bailing Yan, Lei Song, Jia Guo, Yangyang Wang, Liping Peng, Dan Li

**Affiliations:** Department of Respiratory Medicine, The First Hospital of Jilin University, Changchun, China

**Keywords:** COVID-19, chest imaging study, peripheral blood oxygen saturation, observational study, mobile cabin hospital

## Abstract

Majority of patients with 2019 novel coronavirus infection (COVID-19) exhibit mild symptoms. Identification of COVID-19 patients with mild symptoms who might develop into severe or critical illness is essential to save lives. We conducted an observational study in a dedicated make-shift hospital for adult male COVID-19 patients with mild symptoms between February and March 2020. Baseline characteristics, medical history, and clinical presentation were recorded. Laboratory tests and chest computed tomography were performed. Patients were observed until they were either transferred to a hospital for advanced care owing to disease exacerbation or were discharged after improvement. Patients were grouped based on their chest imaging findings or short-term outcomes. A total of 125 COVID-19 patients with mild symptoms were enrolled. Of these, 7 patients were transferred for advanced care while 118 patients were discharged after improvement and showed no disease recurrence during an additional 28-day follow-up period. Eighty-five patients (68.0%) had abnormal chest imaging findings. Patients with abnormal chest imaging findings were more likely to have disease deterioration and require advanced care as compared to those with normal chest imaging findings. Patients with deteriorated outcomes were more likely to have low peripheral blood oxygen saturation and moderately-elevated body temperature. There were no significant differences between patients with deteriorated or improved outcomes with respect to age, comorbidities, or other clinical symptoms (including nasal congestion, sore throat, cough, hemoptysis, sputum production, shortness of breath, fatigue, headache, nausea or vomiting, diarrhea). Abnormal chest imaging findings, low peripheral blood oxygen saturation, and elevated temperature were associated with disease deterioration in adult male COVID-19 patients with mild clinical symptoms.

**Clinical Trial Registration**: https://register.clinicaltrials.gov/prs/app/action/SelectProtocol?sid=S0009RA3&selectaction=Edit&uid=U0003F4L&ts=2&cx=-ajpsbw, identifier NCT04346602.

## Introduction

In December 2019, an outbreak of novel coronavirus pneumonia was reported in Wuhan, China. Subsequently, the outbreak rapidly spread in China and across the world (1). The World Health Organization (WHO) named this new coronavirus as “2019 Novel Coronavirus (2019-nCoV)” (2). This novel coronavirus belongs to the same family as the 2003 severe acute respiratory syndrome coronavirus (SARS-CoV). The human disease caused by this novel coronavirus is referred to as COVID-19, which was declared as a pandemic by WHO (3). As of May 3 2020, the cumulative number of confirmed COVID-19 cases has surpassed 3 million globally, with more than 200,000 deaths reported. The ongoing pandemic has imposed an enormous burden on the society and individual patients. Based on the clinical presentation, laboratory investigations, and imaging findings, patients with COVID-19 can be classified into mild, moderate, severe, or critical illness (4). Several studies have investigated the epidemiology, pathogenesis, and the management of COVID-19. However, most of these studies involved hospitalized patients with moderate or critical illness (5–8). Few studies have investigated patients with mild or moderate illness, despite the fact that more than 80% of patients with COVID-19 exhibit mild or moderate illness (9). It is important to identify patients who have mild initial presenting symptoms, but who are at high risk of developing severe illness. This would help optimize the use of medical resources and reduce the associated morbidity and mortality.

In China, all patients diagnosed with COVID-19 were admitted to a dedicated healthcare facility. This was done to provide centralized care for infected patients and to minimize the risk of community spread of the virus (10). Patients with mild symptoms were admitted to makeshift hospitals referred to as mobile cabin hospitals; these temporary hospitals were typically set up at sports stadiums or convention centers (11, 12). This provided us an opportunity to study the clinical characteristics and short-term outcomes of COVID-19 patients who had mild initial presenting symptoms.

Here, we report the clinical characteristics COVID-19 patients with mild initial presenting symptoms. We further studied the association between the short-term outcomes of these patients and their chest imaging findings and laboratory indices.

## Materials and Methods

### Study Design and Participants

We performed a prospective observational study at a mobile cabin hospital dedicated for treatment of adult male COVID-19 patients with mild symptoms in Wuhan, China between February and March 2020. The study protocol was approved by the hospital ethics committee. Written informed consent was obtained from all subjects prior to their enrolment.

#### Inclusion Criteria

Male patients who had the body temperature remaining below 37.3°C, did not experience the symptoms such as cough, expectoration, dyspnea or diarrhea, did not or only experienced mild throat pain, and reported a positive nucleic acid test over the 1 week prior to admission.

#### Exclusion Criteria

Patients who had the body temperature exceeding 37.3°C and experienced significant dyspnea and/or diarrhea over the 1 week prior to admission.

### Study Protocol

Baseline demographic information and medical history were collected from the subjects before starting treatment on the day of admission. Their clinical presentation was recorded. Laboratory tests included complete blood counts, serum biochemistry, and hepatic and renal function tests. All patients also underwent chest computed tomography (CT).

During their stay at the mobile cabin hospital, patients were closely monitored, including daily physical examination, recording of vital parameters, and pulse oximetry. This was continued until they were either transferred to a hospital for advanced care due to deterioration of their illness or were discharged home after clinical improvement. The criteria for transfer to an advanced care hospital were: (1) exacerbation of any underlying disease, such as hypertension, diabetes, or other illnesses; (2) persistent fever; (3) shortness of breath; (4) oxygen saturation <93% (by pulse oximetry). The criteria for discharge were: (1) resolution of fever for >3 days; (2) improvement in respiratory symptoms; (3) improvement in chest imaging findings; (4) negative nucleic acid test for SARS-CoV-2 in at least two consecutive samples collected more than 24 h apart.

Patients who were discharged were followed up for an additional 28 days and 12 weeks for any signs of disease recurrence.

### Statistical Analysis

Continuous data are presented as mean ± standard deviation or as median and interquartile range (IQR), as appropriate. Categorical data are presented as frequencies and percentages. Patients were grouped based on their chest imaging findings or short-term outcomes. Comparisons between patients with normal or abnormal chest CT findings as well as between patients with deteriorated or improved outcomes were performed using the sum rank test or Chi squared analysis (Fisher exact test), as appropriate. All statistical analyses were performed using SPSS (IBM, USA). *P*-values < 0.05 were considered indicative of statistical significance.

## Results

A total of 309 patients were admitted to our mobile cabin hospital during the study reference period. Of these, 125 patients qualified the inclusion criteria and were enrolled in the study. Complete records pertaining to clinical evaluation and follow-up outcomes were available for all patients. Seven patients were transferred for advanced care due to disease exacerbation. The remaining 118 patients were discharged home and were followed up for 28 days.

All subjects had a recent history of exposure to COVID-19. The median age of patients was 35 years (IQR, 30–49) (Table 1). A large number of patients (82 patients, 65.6%) were current smokers. Most common comorbidities were hypertension (8 patients, 6.4%) and diabetes (8 patients, 6.4%). Two patients (1.6%) had cough and 2 patients (1.6%) had mild shortness of breath. Eight patients (6.4%) had fever. Only one patient (0.8%) had mild diarrhea. A total of 85 (68.0%) patients had abnormal chest CT findings at admission (Table 2). The most common abnormality was ground-glass opacities in the lung (70 patients, 56.0%). A small number of patients had decreased leukocyte counts (6 patients, 4.8%, <4 × 10^9^/L) and lymphocyte counts (21 patients, 16.8%, <1.5 × 10^9^/L). Platelet count was reduced in 15 (12%) patients. There was mild elevation of hepatic enzyme levels. The renal function was intact in all patients.

**Table 1:** Clinical characteristics of the study population.

**Variables**	**All patients (*N* = 125)**
Age, median (IQR), years	35 (30–49)
**Smoking history, N (%)**	
Never smokers	38 (30.4%)
Ex-smokers	5 (4.0%)
Current smokers	82 (65.6%)
Comorbidities, N (%)	14 (14.1%)
COPD	1
Diabetes	8
Hypertension	8
Hepatitis B	1
Fever, N (%)	8 (6.4%)
**Highest temperature during hospital stay, N (%)**	
<37.5^o^C	2 (1.6%)
37.5–38.0^o^C	3 (2.4%)
38.1–39.0^o^C	2 (1.6%)
>39.0^o^C	1 (0.8%)
SpO_2_, median (IQR)	98 (96–100)
**Respiratory symptoms during admission, N (%)**	
Nasal congestion	0 (0%)
Sore throat	0 (0%)
Cough	2 (1.6%)
Hemoptysis	0 (0%)
Sputum production	0 (0%)
Shortness of breath	2 (1.6%)
**Other symptoms, N (%)**	
Fatigue	0 (0%)
Headache	0 (0%)
Nausea or vomiting	0 (0%)
Diarrhea	1 (0.8%)

*COPD, chronic obstructive pulmonary disease; SpO_2_, peripheral oxygen saturation. Non-normally distributed continuous variables were presented as median and interquartile range (IQR): median (IQR). Categorical variables were presented as number and percentage: number (percentage)*.

**Table 2:** Chest imaging findings and laboratory indices.

**Variables**	**All patients (*N* = 125)**
**Chest computed tomography**	
Total abnormalities, N (%)	85 (68.0%)
Ground-glass opacity	70 (56.0%)
Local patchy shadowing	18 (14.4%)
Bilateral patchy shadowing	10 (8.0%)
Interstitial abnormalities	4 (3.2%)
**Complete blood counts**	
Leukocyte count	6.4 (5.3–7.4)
>10 × 10^9^/L	2 (2.1%)
<4 × 10^9^/L	6 (4.8%)
Lymphocyte count	2.0 (1.6–2.3)
<1.5 × 10^9^/L	21 (16.8%)
Platelet count	216 (172–261)
<150 × 10^9^/L	15 (12%)
Hemoglobin level, g/L	145 (139–152)
**Hepatic and renal functions**	
AST > 40 U/L, N (%)	14 (11.2%)
AST > 80 U/L, N (%)	1 (0.8%)
ALT > 40 U/L, N (%)	60 (48.0%)
ALT > 80 U/L, N (%)	16 (12.8%)
TB 17.1 μmol/L, N (%)	7 (0.56%)
Creatinine ≥ 133 μmol/L, N (%)	0 (0%)
**Blood biochemistry**	
Sodium, mmol/L	140.3 (138.4–141.8)
Potassium, mmol/L	4.65 (4.34–5.0)
Chloride, mmol/L	100.3 (99.1–101.5)
CRP ≥ 10 mg/L, N (%)	10 (8.0%)
LDH ≥ 250 U/L, N (%)	1 (0.8%)
CK ≥ 200 U/L, N (%)	4 (0.32)

*AST, aspartate aminotransferase; ALT, alanine aminotransferase; TB, total bilirubin; CRP, C-reactive protein; LDH, lactose dehydrogenase; CK, creatine kinase. Non-normally distributed continuous variables were presented as median and interquartile range (IQR): median (IQR). Categorical variables were presented as number and percentage: number (percentage)*.

### Comparison Between Patients With Normal or Abnormal Chest CT Findings

Out of 125 patients, 40 patients showed normal chest CT findings, while 85 patients exhibited abnormal CT findings (Supplementary Figure 1). Patients with normal chest CT findings were significantly younger than those with abnormal CT findings. There were no significant between-group differences with respect to baseline characteristics, clinical presentation, or medical history (Table 3). Among the laboratory parameters, a greater number of patients in the abnormal CT group had blood leukocyte count lower than 4 × 10^9^/L (Table 4).

**Table 3:** Comparison of baseline characteristics, medical history, and clinical presentation between COVID-19 patients with normal or abnormal chest CT findings.

**Variables**	**Normal CT** **(*N* = 40)**	**Abnormal CT** **(*N* = 85)**	***P*-value**
Age, median (IQR), years	33.5 (27–42)	40 (30–52.5)	0.02
**Smoking history, N (%)**			
Never smokers	15 (37.5%)	23 (27.1%)	0.24
Ex-smokers	2 (5.0%)	3 (3.5%)	0.66
Current smokers	23 (57.5%)	59 (69.4%)	0.19
Comorbidities, N (%)	6/40 (15%)	8/85 (9.4%)	0.60
COPD	1	0	0.32
Diabetes	3	5	1.00
Hypertension	3	5	1.00
Hepatitis B	1	0	0.32
Fever, N (%)	0 (0%)	8 (9.4%)	0.11
**Highest temperature during hospital stay, N (%)**			
<37.5^o^C	0 (0%)	2 (2.4%)	1.00
37.5–38.0^o^C	0 (0%)	3 (3.5%)	0.55
38.1–39.0^o^C	0 (0%)	2 (2.4%)	1.00
>39.0^o^C	0 (0%)	1 (1.2%)	1.00
SpO_2_, median (IQR)	98 (97–100)	97 (95–100)	0.54
Respiratory symptoms during admission, N (%)			1.00
Nasal congestion	0 (0%)	0 (0%)	
Sore throat	0 (0%)	0 (0%)	
Cough	1 (2.5%)	1 (1.2%)	
Hemoptysis	0 (0%)	0 (0%)	
Sputum production	0 (0%)	0 (0%)	
Shortness of breath	1 (2.5%)	1 (1.2%)	
Other symptoms, N (%)			1.00
Fatigue	0 (0%)	0 (0%)	
Headache	0 (0%)	0 (0%)	
Nausea or vomiting	0 (0%)	0 (0%)	
Diarrhea	1 (2.5%)	0 (0%)	

*CT, computed tomography; COPD, chronic obstructive pulmonary disease; SpO_2_, peripheral oxygen saturation; IQR, interquartile range. Non-normally distributed continuous variables were presented as median and interquartile range (IQR): median (IQR). Categorical variables were presented as number and percentage: number (percentage)*.

**Table 4:** Comparison of laboratory indices between COVID-19 patients with normal or abnormal chest CT findings.

**Variables**	**Normal CT** **(*N* = 40)**	**Abnormal CT** **(*N* = 85)**	***P*-value**
**Complete blood counts**			
Leukocyte count	6.1 (5.1–7.3)	6.5 (5.3–7.5)	0.45
>10 × 10^9^/L	2 (5%)	0 (0%)	0.10
<4 × 10^9^/L	0 (0%)	6 (7.1%)	0.02
Lymphocyte count	1.9 (1.7–2.2)	2.0 (1.6–2.5)	0.26
<1.5 × 10^9^/L	4 (10.0%)	17 (20.0%)	0.21
Platelet count	217 (180.8–259.8)	215 (168–268)	0.93
<150 × 10^9^/L	3 (7.5%)	12 (14.1%)	0.38
Hemoglobin level, g/L	145 (139–155)	146 (139–152)	0.60
**Hepatic and renal functions**			
AST > 40 U/L, N (%)	2 (5%)	12 (14.1%)	0.22
AST > 80 U/L, N (%)	0 (0%)	1 (1.2%)	1.00
ALT > 40 U/L, N (%)	19 (47.5%)	41 (48.2%)	1.00
ALT > 80 U/L, N (%)	5 (12.5%)	11 (12.9%)	0.95
TB 17.1 μmol/L, N (%)	2 (5%)	5 (5.9%)	1.00
Creatinine ≥ 133 μmol/L, N (%)	0 (0%)	0 (0%)	1.00
**Blood biochemistry**			
Sodium, mmol/L	139.8 (138.2–141.4)	140.3 (138.5–142.0)	0.54
Potassium, mmol/L	4.6 (4.3–4.9)	4.7 (4.4–5.0)	0.18
Chloride, mmol/L	100.5 (99.1–101.4)	100.1 (99.1–101.5)	0.96
CRP ≥ 10 mg/L, N (%)	0 (0%)	10 (11.8%)	0.10
LDH ≥ 250 U/L, N (%)	0 (0%)	1 (1.2%)	1.00
CK ≥ 200 U/L, N (%)	1 (2.5%)	3 (3.5%)	1.00

*AST, aspartate aminotransferase; ALT, alanine aminotransferase; TB, total bilirubin; CRP, C-reactive protein; LDH, lactose dehydrogenase; CK, creatine kinase. Non-normally distributed continuous variables were presented as median and interquartile range (IQR): median (IQR). Categorical variables were presented as number and percentage: number (percentage)*.

### Comparison Between Patients With Deteriorated or Improved Outcomes

Based on the outcomes (discharged to home or referred for advanced healthcare), the 125 patients were assigned to improved outcome group (118 patients) or deteriorated outcome group (7 patients). None of the 118 patients who were discharged home showed recurrence of their clinical symptoms during the 28-day follow-up period. And in fact, a telephone follow-up at 12 weeks after the patient was discharged from the mobile cabin hospital showed that all patients (125 patients) were in good general condition at that time.

Compared with the improved outcome group, patients in the deteriorated group were more likely to develop fever (Table 5). There were no other significant between-group differences with respect to age, clinical presentation, or medical history.

**Table 5:** Comparison of baseline characteristics, clinical presentation, and medical history between COVID-19 patients with deteriorated or improved outcomes.

**Variables**	**Deteriorated outcome** **(*N* = 7)**	**Improved outcome** **(*N* = 118)**	***P*-value**
Age, median (IQR), years	43 (25–53)	30 (35–49)	0.92
**Smoking history, N (%)**			
Never smokers	2 (28.6%)	36 (30.5%)	1.00
Ex-smokers	0 (0%)	5 (4.2%)	1.00
Current smokers	5 (71.4%)	77 (65.3%)	1.00
Comorbidities, N (%)	1 (14.3%)	13 (11.0%)	1.00
COPD	1 (14.3%)	0 (0)	0.06
Diabetes	0 (0)	8 (6.8)	1.00
Hypertension	0 (0)	8 (6.8)	1.00
Hepatitis B	0 (0)	1 (0.8)	1.00
Fever, N (%)	4 (57.1%)	4 (3.4%)	<0.01
**Highest temperature during hospital stay, N (%)**			
<37.5^o^C	0 (0%)	2 (1.7%)	1.00
37.5–38.0^o^C	1 (14.3%)	2 (1.7%)	0.16
38.1–39.0^o^C	2 (28.6%)	0 (0%)	<0.01
>39.0^o^C	1 (14.3%)	0 (0%)	0.06
SpO_2_, median (IQR)	94 (92–98)	98 (96–100)	0.04
Respiratory symptoms during admission, N (%)			1.00
Nasal congestion	0 (0%)	0 (0%)	
Sore throat	0 (0%)	0 (0%)	
Cough	0 (0%)	2 (1.7%)	
Hemoptysis	0 (0%)	0 (0%)	
Sputum production	0 (0%)	0 (0%)	
Shortness of breath	2 (28.6%)	0 (0%)	
Other symptoms, N (%)			1.00
Fatigue	0 (0%)	0 (0%)	
Headache	0 (0%)	0 (0%)	
Nausea or vomiting	0 (0%)	0 (0%)	
Diarrhea	0 (0%)	1 (0.8%)	

*COPD, chronic obstructive pulmonary disease; SpO_2_, peripheral oxygen saturation. Non-normally distributed continuous variables were presented as median and interquartile range (IQR): median (IQR). Categorical variables were presented as number and percentage: number (percentage)*.

Patients with improved outcomes were less likely to have abnormal chest CT findings and more likely to have had better pulse oximetry readings (Table 6). In addition, the blood leukocyte count in the deteriorated group was significantly higher than that in the improved group.

**Table 6:** Comparison of chest CT findings and laboratory indices between COVID-19 patients with deteriorated or improved outcomes.

**Variables**	**Deteriorated outcome** **(*N* = 7)**	**Improved outcome** **(*N* = 118)**	***P*-value**
**Chest computed tomography scan**			
Total abnormalities, N (%)	7 (100%)	78 (66.1%)	0.10
Ground-glass opacity	7 (100%)	63 (53.4%)	0.02
Local patchy shadowing	6 (85.7%)	12 (10.2%)	<0.001
Bilateral patchy shadowing	5 (71.4%)	5 (4.2%)	<0.001
Interstitial abnormalities	4 (57.1%)	0 (0%)	<0.001
**Laboratory findings**			
**Complete blood counts**			
Leukocyte count	7.9 (6.6–9.5)	6.3 (5.2–7.3)	0.04
>10 × 10^9^/L	1 (14.3%)	1 (0.8%)	0.11
<4 × 10^9^/L	1 (14.3%)	5 (4.2%)	0.30
Lymphocyte count	2.2 (1.6–2.8)	2.0 (1.6–2.3)	0.51
<1.5 × 10^9^/L	1 (14.3%)	20 (17.0%)	1.00
Platelet count	224 (157–303)	215.5 (172.5–261.0)	0.76
<150 × 10^9^/L	1 (14.3%)	14 (11.9%)	1.00
Hemoglobin level, g/L	151 (138–157)	145 (139.0–152.3)	0.52
**Hepatic and renal functions**			
AST > 40 U/L, N (%)	0 (0%)	14 (11.9%)	1.00
AST > 80 U/L, N (%)	0 (0%)	1 (0.8%)	1.00
ALT > 40 U/L, N (%)	2 (28.6%)	48 (40.7%)	0.70
ALT > 80 U/L, N (%)	0 (0%)	16 (13.6%)	0.60
TB 17.1 μmol/L, N (%)	0 (0%)	7 (5.9%)	1.00
Creatinine ≥ 133 μmol/L, N (%)	0 (0%)	0 (0%)	1.00
**Blood biochemistry**			
Sodium, mmol/L	140 (139.1–142.4)	140.3 (138.2–141.8)	0.70
Potassium, mmol/L	5.07 (4.2–5.3)	4.64 (4.4–5.0)	0.26
Chloride, mmol/L	99.0 (98.3–101.8)	100.4 (99.2–101.5)	0.33
CRP ≥ 10 mg/L, N (%)	1 (14.3%)	9 (7.6%)	0.45
LDH ≥ 250 U/L, N (%)	0 (0%)	1 (0.8%)	1.00
CK ≥ 200 U/L, N (%)	0 (0%)	4 (3.4%)	1.00

*CT, computed tomography; AST, aspartate aminotransferase; ALT, alanine aminotransferase; TB, total bilirubin; CRP, C-reactive protein; LDH, lactose dehydrogenase; CK, creatine kinase. Non-normally distributed continuous variables were presented as median and interquartile range (IQR): median (IQR). Categorical variables were presented as number and percentage: number (percentage)*.

In order to further evaluate the effects of body temperature, WBC, peripheral blood oxygen saturation, and CT changes on the prognosis, we analyzed the correlation (by Spearman Rank Correlation) between the prognosis and various indicators, and the results confirmed that body temperature, leukocyte counts, and peripheral blood oxygen saturation (SpO_2_) are closely correlated with the prognosis. CT changes showed some correlation with the prognosis, which however was not statistically significant (Supplementary Table 1).

## Discussion

The ongoing pandemic of COVID-19 has imposed an enormous burden on the societies and individuals across the world (13–15). Early studies have categorized the clinical spectrum of COVID-19 into mild, moderate, severe, or critical illness (4). More than 80% of patients tend to recover with supportive care (9). Fewer than 20% of patients require hospital admission for advanced healthcare. Previous studies have identified several risk factors for severe or critical illness; these include age, presence of comorbidities (such as hypertension, obesity, and diabetes), and certain laboratory indices (6, 8, 16–18). However, young patients with no comorbid conditions who initially exhibit mild illness may rapidly develop severe or critical illness, and may even die (19). Therefore, it is important to identify patients who have mild symptom at onset, but who are at a high-risk of developing critical illness. This can help optimize the use of healthcare resources and facilitate early intervention for high-risk patients.

COVID-19 may affect multiple organ systems in the body; however, it most commonly causes pneumonia (20). Respiratory involvement is also the most common cause of death of patients with COVID-19 (21). Therefore, we first investigated the chest imaging findings in COVID-19 with mild clinical symptoms. Our results indicate that a sizable proportion of patients (68.0%) with mild clinical symptoms may have abnormal chest imaging findings. Ground-glass opacity was the most common chest imaging abnormality. We further compared the differences between patients with improved or deteriorated outcomes during the stay at the mobile cabin hospital. Patients with deteriorated outcomes were significantly more likely to exhibit abnormal chest imaging findings than patients who had improved outcomes. Very few patients in our cohort had cough (2 patients) or shortness of breath (2 patients), even though a majority of patients (85, 68.0%) had abnormal chest imaging findings. This is consistent with a previous report that documented typical abnormal chest imaging findings in asymptomatic patients with COVID-19 (22). Therefore, we recommend chest imaging study of all patients with COVID-19, including those with mild symptoms. This may help identify patients who are at high risk of developing severe illness.

Chest imaging helps in the direct evaluation of pulmonary involvement in patients with COVID-19. However, chest imaging, especially CT scan, entails the risk of radiation exposure. It is also inconvenient to frequently perform repeat chest imaging. Peripheral oxygen saturation measurement is another method to indirectly assess the pulmonary ventilation and oxygenation status (23). Moreover, it is a convenient and safe procedure. In our study, patients with deteriorated outcomes were significantly more likely to have low peripheral oxygen saturation (Supplementary Figure 2) than patients with improved outcomes. This suggested that monitoring of peripheral oxygen saturation is a useful method for identification of patients who may develop severe illness. Early initiation of oxygen therapy may reverse or save their lives.

In our study, patients with deteriorated outcomes were more likely to have moderately elevated temperature and elevated blood leukocyte count. Fever is the most common presenting feature of COVID-19 (24). Studies have shown that SARS-CoV may attack leukocytes resulting in reduced leukocyte counts (25, 26). Further studies are required to assess the predictive value of body temperature and leukocyte count in COVID-19 patients with mild symptoms.

Several studies have identified comorbid conditions such as hypertension, diabetes, and chronic pulmonary disease as risk factors for severe and critical COVID-19 illness (6, 8, 16–18). In our study, we did not observe any significant association of comorbidities with chest imaging findings or outcomes. Age was also found to be a risk factor for increased morbidity and mortality in COVID-19 patients (27). In our study, older patients were more likely to have abnormal chest CT findings. However, age was not associated with the short-term outcome. We believe that the risk factors may differ depending on the initial presentation (mild or severe symptoms) of COVID-19 patients.

COVID-19 may impair the functioning of multiple organs and systems in the body. In addition to lung injury, liver and kidney are also frequently affected in these patients (28). Some of the patients in our cohort exhibited mild elevation in liver enzyme levels; however, there was no significant difference between patients with deteriorated or improved outcomes in this respect. None of our patients showed any sign of kidney injury.

The single-center scope of our study and the relatively small sample size are some of the limitations of our study. We were only able to study adult male patients, since our mobile cabin hospital was exclusively dedicated for hospitalization of adult male COVID-19 patients. Lastly, this was an observational study; therefore, we could not control the treatment modalities, such as oxygen supplementation or administration of certain traditional Chinese medicines.

In summary, we observed an association of chest imaging findings, peripheral blood oxygen saturation, and body temperature with disease deterioration in adult male COVID-19 patients with mild clinical symptoms. Close monitoring of these indices may facilitate identification of patients who are high risk of developing severe or critical illness. This can help optimize the use of healthcare resources and facilitate early interventions to reduce morbidity and mortality in high-risk patients.

## Data Availability Statement

The original contributions presented in the study are included in the article/Supplementary Material, further inquiries can be directed to the corresponding authors.

## Ethics Statement

The studies involving human participants were reviewed and approved by ethics committee of First Hospital of Jilin University. The patients/participants provided their written informed consent to participate in this study.

## Author Contributions

LP and DL conceived, designed the study, and had full access to all the data in the study and take responsibility for the integrity of the data and the accuracy of the data analysis. BY and YW conducted the primary analysis and prepared the first draft of the manuscript. LS and JG reviewed the draft for intellectual content. All authors contributed to the article and approved the submitted version.

## Conflict of Interest

The authors declare that the research was conducted in the absence of any commercial or financial relationships that could be construed as a potential conflict of interest.
