# Supplementary material for: Association Between Clinical Characteristics and Short-Term Outcomes in Adult Male COVID-19 Patients With Mild Clinical Symptoms: A Single-Center Observational Study
Source: Front Med (Lausanne). 2021 Jan 5;7:571396. doi: 10.3389/fmed.2020.571396 (PMC7813813; doi:10.3389/fmed.2020.571396)
Supplement: Supplementary file 1 [file Data_Sheet_1.docx]

**Association between clinical characteristics and short-term outcomes in adult male COVID-19 patients with mild clinical symptoms: a single-center observational study**

Bailing Yan, Lei Song, Jia Guo, Yangyang Wang, Liping Peng^*^, Dan Li^*^

Department of Respiratory Medicine, The First Hospital of Jilin University, Changchun, China, 130021.

^*^Contributed equally

^*^ Corresponding authors: Liping Peng (E-mail:[plp640317@163.com](mailto:plp640317@163.com)) and Dan Li (E-mail: li_dan@jlu.edu.cn), Department of Respiratory Medicine, The First Hospital of Jilin University, 1Xinmin Street, Changchun, 130021, Jilin Province, People’s Republic of China.

**Supplementary information**

**Imaging scoring method**

Depending on the extent of involvement, a single lobe was to be classified as either no involvement (0%), mild involvement (1%-25%), moderate involvement (26%-50%), severe involvement (51%-75%), or very severe involvement (76%). %-100%).

The score was 0, 1, 2, 3 and 4 for a lung lobe with no involvement, mild involvement, moderate involvement, severe involvement, and very severe involvement, respectively. The total score of lung involvement was the sum of the scores for the 5 lung lobes (possible range of overall score: 0-20). Based on the numerical value of the total lung score, patients were classified on CT as in either mild (0-5 points), progressive (6-10 points), severe (11-15 points), or critical (≥16 points) stage[1].

The total lung scores of the COVID-19 patients included in this study ranged from 0 to 13, including 40, 78, 6, 1 and 0 patients with the scores of ≤ 5, 6-10, 11-15, and ≥16, respectively.

In order to further evaluate the effects of body temperature, WBC, peripheral blood oxygen saturation, and CT changes on the prognosis, we analyzed the correlation (by Spearman Rank Correlation) between the prognosis and various indicators, and the results confirmed that body temperature, leukocyte counts, and peripheral blood oxygen saturation (SpO_2_) are closely correlated with the prognosis. CT changes showed some correlation with the prognosis, which however was not statistically significant. (Table. S1).

Table. S1. Spearman’s correlations of outcomes with temperature, SpO2, leukocyte counts and chest image scores respectively

|  | temperature | | SpO_2_ | | leukocyte counts | | chest image scores | |
| --- | --- | --- | --- | --- | --- | --- | --- | --- |
|  | r | P | r | P | r | P | r | P |
| outcomes | 0.618 | 0.000 | -0.365 | 0.000 | 0.180 | 0.045 | 0.144 | 0.109 |


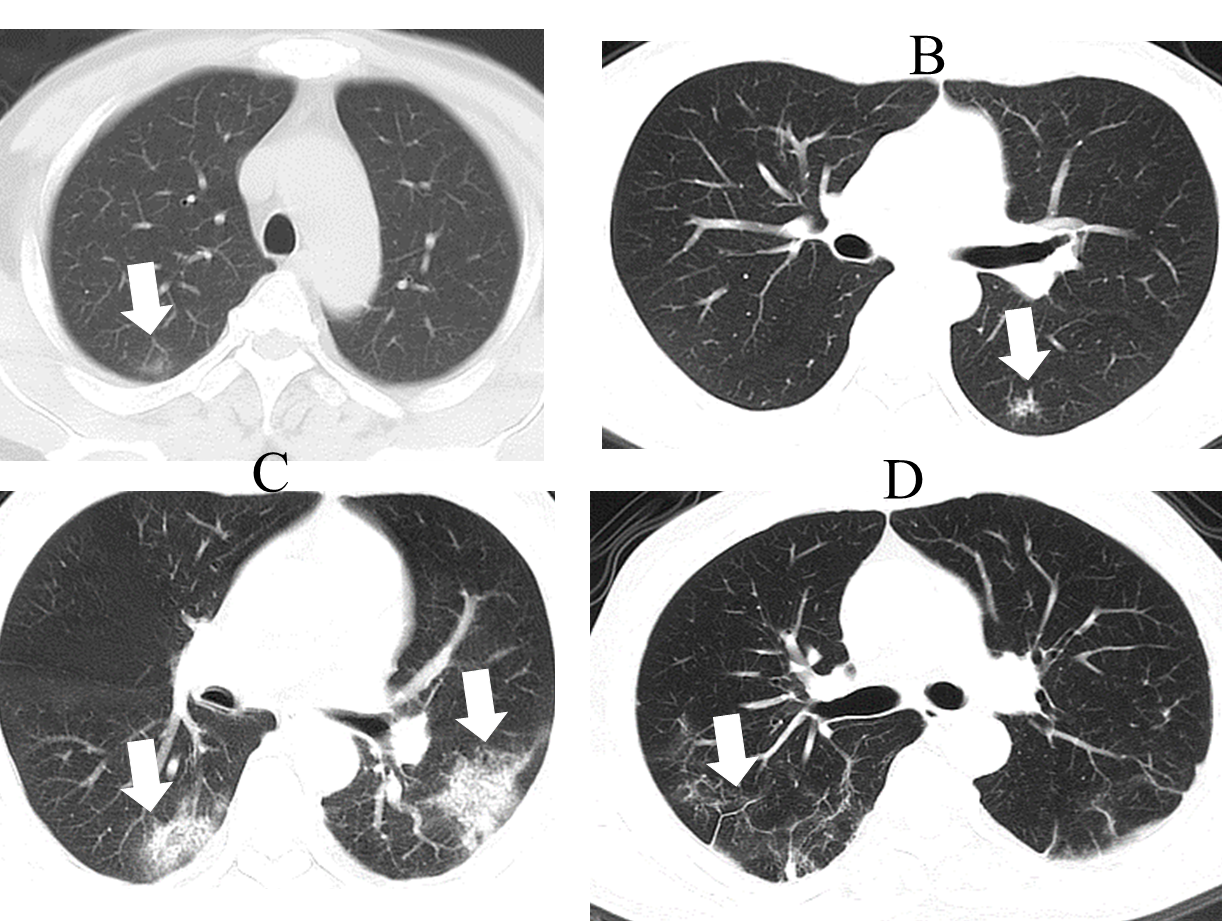


A

Fig.S1. Abnormal chest CT findings in COVID-19 patients. A: Ground-glass opacity; B: Local patchy shadowing: C: Bilateral patchy shadowing; D: Interstitial abnormalities.


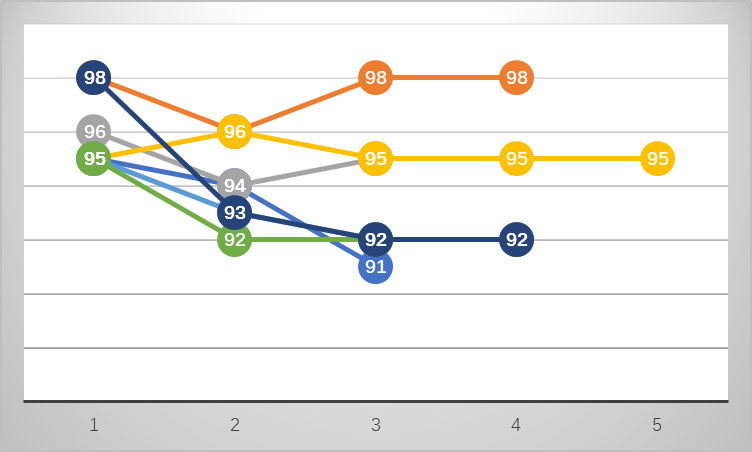


d1 d2 d3 d4 d5

Fig. S2. Dynamical changed of SpO2 of 7 patients with deteriorated outcomes.

**Reference**

1. Chung, M., et al., *CT Imaging Features of 2019 Novel Coronavirus (2019-nCoV).* Radiology, 2020. **295**(1): p. 202-207.
